# Curcuminoid supplementation for vasomotor symptoms in postmenopausal women: a pilot, randomized, double-blind, placebo-controlled proof-of-concept trial

**DOI:** 10.1186/s12906-026-05358-7

**Published:** 2026-03-24

**Authors:** Jinjutha Buntoonprayuk, Kitti Chattrakulchai, Orawin Vallibhakara, Pimpun Prasanchit, Sakda Arj-Ong Vallibhakara

**Affiliations:** 1 Department of Obstetrics and Gynaecology, Faculty of Medicine Ramathibodi Hospital, Mahidol University, Bangkok, Thailand; 2 Menopause Unit, Reproductive Endocrinology and Infertility Division, Department of Obstetrics and Gynaecology, Faculty of Medicine Ramathibodi Hospital, Mahidol University, Bangkok, Thailand; 3 Reproductive Endocrinology and Infertility Division, Department of Obstetrics and Gynaecology, Faculty of Medicine Ramathibodi Hospital, Mahidol University, Bangkok, Thailand; 4 Interdisciplinary Studies and Lifelong Education (I-Grad), Faculty of Public Health, Mahidol University, Bangkok, Thailand

**Keywords:** Curcuminoids, Menopause, Vasomotor symptoms, Hot flashes, Quality of life, Randomized controlled trial, Pilot study, Pilot study

## Abstract

**Background:**

Vasomotor symptoms are common during menopause and can impair quality of life. Although hormone therapy is effective, concerns about risks and adverse effects contribute to limited utilization, highlighting the need for non-hormonal options. Curcuminoids have antioxidant and anti-inflammatory properties and may improve vasomotor symptoms.

**Methods:**

This 12-week, randomized, double-blind, placebo-controlled pilot proof-of-concept trial evaluated the preliminary efficacy of curcuminoids (1000 mg/day) versus placebo in healthy postmenopausal women (41–65 years; *n* = 40). The prespecified primary endpoint was the between-group difference in change in weekly hot flash frequency from baseline to Week 12. Secondary outcomes included changes in Menopause Rating Scale II (MRS II) and Menopause-Specific Quality of Life (MENQOL) scores. Safety and tolerability were assessed throughout.

**Results:**

All participants completed the intervention with high adherence and no serious adverse events. At Week 12 (primary endpoint), the between-group difference in change in weekly hot flash frequency from baseline was − 7.35 episodes/week (95% CI − 10.29 to − 4.41; *p* < 0.001), favoring curcuminoids; results were concordant using the Hodges–Lehmann estimate (− 6.00; 95% CI − 10.00 to − 4.00; *p* < 0.001). A significant between-group difference was also observed at Week 6. No statistically significant between-group differences were observed at Week 12 for total MENQOL or total MRS II, although the MENQOL vasomotor domain improved at Week 6 and showed supportive evidence in baseline-adjusted sensitivity analysis at Week 12.

**Conclusion:**

In this pilot proof-of-concept randomized trial, curcuminoid supplementation significantly reduced weekly hot flash frequency compared with placebo and showed a preliminary signal toward improved vasomotor-related quality of life, particularly at Week 6, with good tolerability. These preliminary, hypothesis-generating findings require confirmation in larger, adequately powered randomized trials, ideally incorporating biomarker endpoints.

**Trial registration:**

Thai Clinical Trials Registry (TCTR) TCTR20241210002 (retrospectively registered).

**Supplementary Information:**

The online version contains supplementary material available at 10.1186/s12906-026-05358-7.

## Introduction

Menopause, defined as the absence of menstruation for 12 consecutive months, marks the permanent cessation of ovarian function [1]. Over 80% of women report menopausal symptoms, and approximately one-third experience severe symptoms that can significantly affect their daily lives and overall quality of life [2, 3]. Vasomotor symptoms (VMS), comprising hot flashes and night sweats, are frequently accompanied by sleep disturbances and anxiety. The unpredictable nature of these episodes often disrupts daily activities and leads to social withdrawal [4]. Although VMS typically persist for one to six years, 10–15% of women may remain symptomatic for more than 15 years [5]. Hormone therapy remains the first-line treatment; however, concerns regarding potential risks contribute to limited clinical uptake, highlighting the need for non-hormonal options [6–8]. Consequently, the American College of Obstetricians and Gynecologists (ACOG) recommends exploring alternative VMS treatments [6]. Turmeric (*Curcuma longa*) contains curcuminoids, of which curcumin is the major constituent, with established antioxidant and anti-inflammatory properties [9]. Turmeric rhizomes typically contain 2–6% curcuminoids by weight [10], and curcumin is recognized by the U.S. FDA as generally recognized as safe (GRAS) and is widely used as a dietary supplement [11]. Importantly, the rationale for human testing is supported by experimental (in vitro and animal) evidence rather than traditional use alone.

In ovariectomized (OVX) rat models, curcumin and its major metabolite, tetrahydrocurcumin (THC), reduced tail skin temperature, which is a validated proxy for vasomotor instability, with efficacy comparable to 17β-estradiol despite no restoration of serum estrogen or uterine weight [12]. Across experimental models, curcuminoids have been reported to attenuate inflammatory signaling and oxidative stress and to influence central pathways relevant to thermoregulation [13–18]. Together, these findings provide a biologically plausible basis to investigate standardized curcuminoid supplementation in postmenopausal women. In parallel, menopause-associated estrogen decline impairs endogenous antioxidant defenses, elevating oxidative stress and inflammation, which are linked to VMS and cardiometabolic complications [19–23]. Because VMS involves vasodilation and increased metabolic activity that may increase reactive oxygen species generation [24, 25], antioxidant strategies have attracted interest.

Emerging clinical evidence suggests that curcumin supplementation may improve endothelial function and reduce hot flash frequency [26]. However, existing studies remain heterogeneous in dose, formulation, and duration; a recent meta-analysis highlighted wide dose variability (80–1000 mg/day), underscoring the need for standardized protocols [15]. Moreover, double-blind, placebo-controlled trials of standardized curcuminoid monotherapy are limited, particularly in Southeast Asian populations where dietary and genetic factors may modulate response.

Therefore, we conducted a randomized, double-blind, placebo-controlled pilot proof-of-concept trial to evaluate the preliminary efficacy of 12-week curcuminoid supplementation for VMS. The prespecified primary endpoint was the between-group difference in the change in weekly hot flash frequency from baseline to Week 12. Secondary outcomes included between-group differences in changes in Menopause Rating Scale II (MRS II) and Menopause-Specific Quality of Life (MENQOL) scores.

## Materials and methods

### Study design and setting

This study was a 12-week, randomized, double-blind, placebo-controlled pilot proof-of-concept trial. The study was conducted at the Menopause Clinic and the Outpatient Department of Obstetrics and Gynecology, Ramathibodi Hospital, Mahidol University, Bangkok, Thailand. The trial was reported in accordance with the CONSORT statement guidelines, including the checklist and flow diagram [27].

### Participants

Participants were recruited between August 28, 2024, and January 3, 2025. Eligible individuals were postmenopausal women aged 41–65 years with natural or surgical menopause (confirmed by clinical criteria or follicle-stimulating hormone (FSH) > 40 mIU/mL). Eligibility was defined as the presence of vasomotor symptoms, defined as a score of ≥ 1 (mild or greater) on Item 1 (“hot flashes, sweating”) of the Menopause Rating Scale II (MRS II) at screening. While other menopausal symptoms were assessed using the MRS II and MENQOL at baseline and follow-up, they were not required for inclusion. Participants also needed to be able to read and communicate in Thai and to provide written informed consent.

Exclusion criteria included: indication for mandatory hormonal therapy (e.g., premature hypoestrogenism, osteoporosis), use of hormonal therapy, hormonal contraception, SSRIs, or SNRIs within the previous three months; known hypersensitivity to curcuminoids; severe psychiatric disorders; uncontrolled underlying diseases (e.g., autoimmune disorders, thyroid dysfunction, or active malignancy); smoking; alcohol intake > 10 mL/day; caffeine consumption > 500 mg/day; concurrent use of antioxidant supplements; and contraindications for curcuminoids such as biliary obstruction or gallstones. These criteria aimed to minimize confounding and ensure participant safety and reduce clinical variability. Participants were withdrawn if they developed hypersensitivity to the investigational product, initiated hormonal therapy, began other treatment for menopausal symptoms, or started antioxidant supplementation during the trial.

### Interventions

Participants in the intervention group received curcuminoid capsules (turmeric extract) manufactured and supplied by the Government Pharmaceutical Organization (GPO), Thailand, for research use. Each capsule contained 294.12 mg of turmeric extract, equivalent to 250 mg of total curcuminoids; excipients included colloidal silicon dioxide and magnesium stearate.

Participants took two capsules twice daily (totaling 1,000 mg curcuminoids/day). The extract was derived from Curcuma longa L. rhizomes using 96% ethanol extraction and was standardized to contain 82.0%–88.0% total curcuminoids (comprising curcumin, demethoxycurcumin, and bisdemethoxycurcumin), with residual ethanol content ≤ 5,000 ppm. Quality control measures included strict limits for heavy metals and microbial contamination. The control group received matched placebo capsules, identical in appearance and excipients but without turmeric extract. Both active and placebo products were manufactured and quality-assured by the GPO. Use of other non-hormonal therapies for vasomotor symptoms (SSRIs/SNRIs, gabapentin, or antioxidant supplements) was prohibited; other stable medications were permitted if not expected to affect outcomes.

### Outcomes

The prespecified primary clinical endpoint for preliminary efficacy was the between-group difference in the change in weekly hot flash frequency from baseline to Week 12. Vasomotor symptoms were assessed using a daily VMS diary in which participants recorded the number of hot flashes each day. Diary entries were aggregated into weekly totals over three assessment periods: the 7 days prior to randomization (baseline) and the 7-day periods preceding the Week 6 and Week 12 visits. Week 12 was prespecified as the primary endpoint time point; Week 6 was included as a secondary time point.

Secondary clinical outcomes included between-group differences in changes from baseline in total Menopause Rating Scale II (MRS II) and total Menopause-Specific Quality of Life Questionnaire (MENQOL) scores assessed at baseline, Week 6, and Week 12. The MRS II is an established 11-item instrument designed to evaluate the severity of menopausal symptoms across somato-vegetative, psychological, and urogenital domains using a 5-point Likert-type scale [28]. Each question is scored from 0 (no symptoms) to 4 (most severe symptoms), yielding a cumulative score between 0 and 44, where higher totals reflect greater symptom severity. Its Thai version shows acceptable psychometric properties (69.3% sensitivity, 76.9% specificity) for Thai postmenopausal women [29]. The Menopause-Specific Quality of Life Questionnaire (MENQOL), a recognized 29-item instrument, was used to assess quality of life across four domains (vasomotor, psychosocial, physical, and sexual) with a 7-point Likert-type scale [30]. The Thai version demonstrates strong reliability, with a Cronbach’s alpha of 0.85 and strong content validity (CVI = 0.94) [31].

### Trial conduct and safety outcomes

Consistent with the pilot proof-of-concept design, trial conduct/process indicators were monitored descriptively as supportive measures to inform the design of a future adequately powered confirmatory trial. These indicators included recruitment yield, retention, protocol compliance (diary completion and visit attendance), and intervention adherence (returned capsule counts and participant interviews). Safety and tolerability were assessed throughout the study period via adverse event reporting and clinical evaluation.

### Randomization and blinding

Participants were randomly allocated in a 1:1 ratio to receive either curcuminoid or placebo using a computer-generated randomization sequence. Block randomization, utilizing a consistent block size of four, was implemented to achieve balanced allocation between groups. Group assignment was concealed using sequentially numbered, opaque, sealed envelopes (SNOSE) prepared by an independent co-investigator who had no role in enrolment, follow-up, outcome assessment, data collection, or analysis. A designated study nurse, who was not involved in participant follow-up, outcome assessment or data collection, opened the next envelope in sequence and recorded the assigned group alongside the participant’s study ID. Consequently, blinding was preserved for participants, clinical staff involved in follow-up, outcome assessors, and data collectors. The data analyst remained blinded to group allocation until the final analysis.

### Sample size calculation

This study was designed as a pilot proof-of-concept randomized controlled trial to evaluate a preliminary efficacy signal and to obtain initial estimates of effect size and variability to inform the design of a future adequately powered confirmatory trial. The target sample size was set a priori for the prespecified primary endpoint using a planning approach informed by the variability and plausible effect magnitude reported in a comparable randomized trial (Ataei-Almanghadim et al. [32]). This planning approach was intended to support detection of only relatively large signals for the purpose of initial estimation and future trial design, not for confirmatory hypothesis testing. Allowing for up to 20% attrition, we planned to enroll 40 participants (20 per group). We acknowledge that this pilot sample may be underpowered to detect small-to-moderate between-group differences; accordingly, efficacy estimates should be interpreted as preliminary and hypothesis-generating.

### Statistical analysis

All analyses were conducted using Stata version 16.0 (StataCorp, College Station, TX, USA) under the intention-to-treat principle. As there was no loss to follow-up and no missing data, all randomized participants were analyzed without imputation. Consistent with the pilot proof-of-concept design, analyses emphasize estimation (effect estimates with 95% confidence intervals [CIs]); p-values are reported as a conventional reference (*p* < 0.05). No formal multiplicity adjustment was applied for secondary outcomes across domains and time points; therefore, secondary-outcome p-values are nominal and interpreted as exploratory and hypothesis-generating, particularly when *p* > 0.01.

Between-group comparisons at each follow-up prioritized change-from-baseline analysis. Normality of change scores was assessed using the Shapiro–Wilk test. Approximately normally distributed outcomes were summarized as mean (SD) and compared using two-sample t-tests (Welch’s t-test when variances were unequal), reporting mean differences with 95% CIs. Non-normally distributed outcomes were summarized as median (IQR) and compared using the Wilcoxon rank-sum test; between-group differences were summarized using Hodges–Lehmann (HL) estimates with 95% CIs. Effect sizes were reported to aid interpretability (Hedges’ g for mean-based comparisons; rank-biserial correlation [*r*_rb_] for Wilcoxon comparisons) and were interpreted cautiously given the pilot sample size.

Absolute values at each time point are presented descriptively; within-group changes were assessed using paired t-tests or Wilcoxon signed-rank tests as appropriate. Baseline-adjusted sensitivity analyses were conducted at Week 12 for the prespecified primary outcome and the secondary outcomes (MENQOL and MRS II) (Additional file 1: Table S2). Weekly hot flash frequency was analyzed using Poisson regression with a log link (incidence rate ratios [IRRs] with 95% CIs), and MENQOL and MRS II outcomes were analyzed using ANCOVA (adjusted mean differences with 95% CIs). These sensitivity analyses are supportive and do not replace the prespecified primary change-from-baseline analysis.

### Study flow and compliance monitoring

**Fig. 1:**
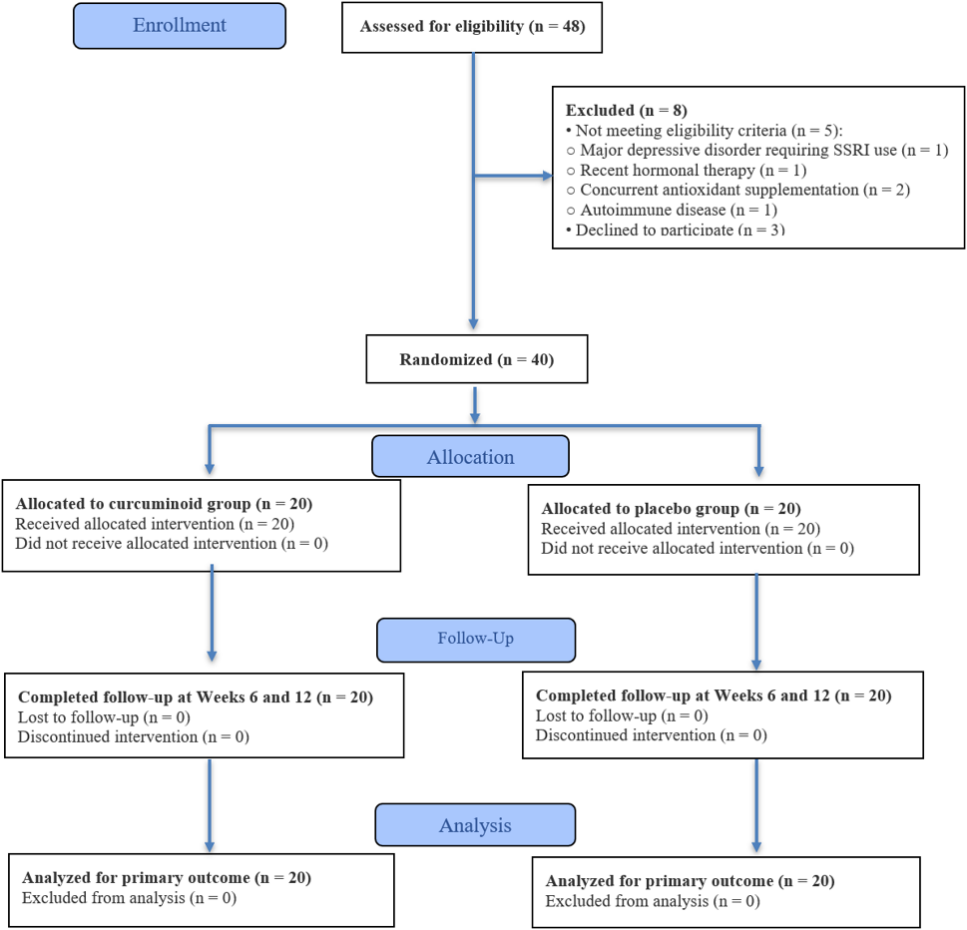
CONSORT flow diagram showing participant screening, randomization, allocation, follow-up, and analysis. A total of 48 postmenopausal women were assessed for eligibility; 8 were excluded (reasons shown). Forty participants were randomized (*n* = 20 per group). No participants discontinued the intervention or were lost to follow-up; all randomized participants were included in the analysis in this pilot proof-of-concept trial

As illustrated in Fig. 1 and 40 eligible participants were randomized (1:1) to receive curcuminoids (*n* = 20) or placebo (*n* = 20). All participants completed a daily VMS diary starting one week prior to intervention. Weekly telephone follow-ups were conducted to reinforce adherence and monitor compliance. In-person visits at Weeks 6 and 12 included collection of VMS diaries, completion of the MRS II and MENQOL questionnaires, and assessment of adverse events. Adherence to the assigned intervention was assessed through participant interviews and returned capsule counts.

### Ethical considerations and trial registration

The study protocol, including all prespecified outcomes, assessment time points, and the primary analysis plan, was approved by the Ethics Committee of the Faculty of Medicine, Ramathibodi Hospital, Mahidol University (COA No. MURA2024/379; approved 28 May 2024) prior to participant enrolment, and all participants provided written informed consent. The trial was registered with the Thai Clinical Trials Registry (TCTR20241210002; retrospectively registered) and is publicly accessible. No changes or amendments were made to the prespecified outcomes, time points, or the analysis plan after enrolment began, and the registry entry was reviewed to confirm consistency of the registered primary outcome and target sample size with this manuscript. Furthermore, the statistical analysis plan was finalized prior to finalizing the dataset, and the lead analyst remained blinded to group allocation until completion of the primary analysis.

### Protocol availability and data sharing

The study protocol, statistical analysis plan, and datasets generated and analyzed during the current study are available from the corresponding author upon reasonable request.

## Results

### Participant flow

A total of 48 postmenopausal women were assessed for eligibility. Eight participants were excluded: five did not meet the eligibility criteria (major depressive disorder requiring SSRI use [*n* = 1], recent hormonal therapy [*n* = 1], concurrent antioxidant supplementation [*n* = 2], and autoimmune disease [*n* = 1]), and three declined to participate. The remaining 40 participants were randomized equally to the curcuminoid (*n* = 20) and placebo (*n* = 20) groups. No participants discontinued the intervention or were lost to follow-up; all randomized participants were included in the analysis (Fig. 1).

### Baseline characteristics

Baseline demographic and clinical characteristics were comparable between the two groups (Table 1). Although baseline differences were not statistically significant, baseline weekly hot flash frequency (15.55 vs. 13.30 episodes/week) and total MENQOL scores (89.80 vs. 83.70) were numerically higher in the curcuminoid group (Additional file 1: Tables S1A and S1C). Primary inference emphasized between-group comparisons of change from baseline, and supportive baseline-adjusted sensitivity analyses at Week 12 are reported in Additional file 1: Table S2.

**Table 1:** Baseline characteristics of participants

**Variable**	**Curcuminoid (** ***n*** **=20)**	**Placebo** (*n*=20)	***p*** **-value**
Age (years), mean (SD)	51.60 (4.63)	54.30 (4.39)	0.070
BMI (kg/m^2^), mean (SD)	27.30 (5.03)	27.40 (4.63)	0.991
Age at menopause (years), mean (SD)	49.00 (3.84)	49.50 (3.44)	0.651
Natural menopause, *n* (%)	18 (90.00)	19 (95.00)	0.550
Parity, median (IQR)	2.00 (1.00−3.00)	2.00 (2.00−3.00)	0.570
Sexually active, *n* (%)	17 (85.00)	14 (70.00)	0.250
Comorbidity, *n* (%)	7 (35.00)	9 (45.00)	0.740
Bachelor's Degree or higher, *n* (%)	13 (65.00)	9 (45.00)	0.210
Income ≥ 15,000 THB/month, *n* (%)	9 (45.00)	12 (60.00)	0.340
Primary Income earner, *n* (%)	12 (60.00)	11 (55.00)	0.760
Urban residence, *n* (%)	15 (75.00)	13 (65.00)	0.490

Values are presented as mean (SD), median (IQR), or *n* (%), as appropriate

*BMI* Body mass index

*p*-values were calculated using independent t tests or Mann–Whitney U tests for continuous variables, and chi-square tests or Fisher’s exact tests for categorical variables, as appropriate

### Primary outcome: weekly hot flash frequency (baseline to Week 12)

At Week 12 (primary endpoint), the between-group difference in change in weekly hot flash frequency from baseline was − 7.35 episodes/week (95% CI − 10.29 to − 4.41; *p* < 0.001), favoring curcuminoids (Table 2). Given the pilot design, effect-size estimates (Tables 2, 3 and 4) are provided for estimation and should be interpreted with caution. Consistent with the prespecified non-parametric approach for non-normally distributed change scores, results were concordant using the Hodges–Lehmann estimate (− 6.00 episodes/week; 95% CI − 10.00 to − 4.00; *p* < 0.001) (Table 2). Absolute weekly hot flash frequency at each time point is presented in Table 2 for clinical context.

**Table 2:** Weekly hot flash frequency and change from baseline to weeks 6 and 12

**Outcome / Timepoint**	**Curcuminoid ** (***n***=20)	**Placebo **(***n***=20)	**Between-group difference [95% CI]**	***p*** **-value**	**Effect Size**
*Change from Baseline* *(episodes/week)*					
Week 12 (Primary Endpoint)					
Mean (SD)	−10.35 (5.90)	−3.00 (2.43)	−7.35 [−10.29 to −4.41]^a^	<0.001*	g = −1.60
Median (IQR)	−8.50 (−14.50, −6.00)	−2.00 (−4.25, −1.00)	−6.00 [−10.00 to −4.00]^b^	<0.001*	*r*_rb_ = −0.81
Week 6					
Mean (SD)	−9.40 (5.10)	−2.45 (2.19)	−6.95 [−9.50 to −4.40]^a^	<0.001*	g = −1.74
Median (IQR)	−9.00 (−12.25, −5.75)	−2.50 (−4.00, −1.75)	−7.00 [−9.00 to −4.00]^b^	<0.001*	*r*_rb_ = −0.82
*Absolute Frequency (episodes/week)*					
Baseline					
Mean (SD)	15.55 (8.30)	13.30 (6.01)	—	—	—
Median (IQR)	16.00 (9.25, 21.25)	10.00 (8.75, 16.25)	2.00 [−3.00 to 7.00]ᵇ	0.400	—
Week 6					
Mean (SD)	6.15 (4.17)	10.85 (5.36)	—	—	—
Median (IQR)	6.00 (2.00, 8.50)	10.00 (7.00, 13.00)	−4.00 [−7.00 to −1.00]ᵇ	0.003*	*r*_rb_ = −0.54
Week 12					
Mean (SD)	5.20 (3.76)	10.30 (5.31)	—	—	—
Median (IQR)	4.00 (2.00, 8.00)	9.00 (7.00, 12.00)	−5.00 [−7.00 to −2.00]ᵇ	0.002*	*r*_rb_ = −0.58

Values are presented as mean (SD) and median (IQR). Change scores were calculated as follow-up minus baseline; negative values indicate a reduction in weekly hot flash frequency. For rows summarized as median (IQR), the between-group difference reported corresponds to the Hodges–Lehmann (HL) estimate (Curcuminoid − Placebo) with 95% CI, not a simple difference in medians. For absolute frequency, mean (SD) rows are descriptive; between-group differences and p-values are reported for the median (IQR) rows. “—” indicates not estimated/not applicable. Effect sizes are provided for estimation: Hedges’ g is based on mean change scores; *r*_*rb*_ is the U-based rank-biserial correlation for Wilcoxon rank-sum comparisons. Effect sizes are not reported at baseline and should be interpreted cautiously given the sample size

*Abbreviations*: *SD* Standard deviation, *IQR* Interquartile range, *CI* Confidence interval, *HL* Hodges–Lehmann, *r*_*rb*_ rank-biserial correlation

^a^Mean differences (Curcuminoid − Placebo) with 95% CIs and p-values for change scores were obtained from Welch’s two-sample t-test (supportive)

^b^HL estimates (Curcuminoid − Placebo) with 95% CIs and p-values were obtained from the Wilcoxon rank-sum test

^*^p < 0.05. Negative between-group differences indicate fewer hot flash episodes or a greater reduction in the curcuminoid group

### Secondary outcomes: MRS II

Both groups improved in MRS II scores over time; however, no statistically significant between-group differences were observed at Week 12 for the MRS II total score or any domain (Table 3, Panels A and B). For the total score (change from baseline), the Hodges–Lehmann estimate at Week 12 was − 2.00 (95% CI − 5.00 to 1.00; *p* = 0.121) (Table 3, Panel B).

**Table 3:** Changes in menopause rating scale II (MRS II) scores from baseline to weeks 6 and 12

**Outcome / Timepoint**	Curcuminoid (***n***=20)	**Placebo** (*n*=20)	**Difference [95% CI]**	***p*** **-** **value**	**Effect size**
*Panel A: presents mean (SD) with Welch’s t-test and Hedges’ g. *					
Somato-vegetative domain score					
Change at Week 12	−2.25 (1.86)	−2.10 (1.45)	−0.15 [−1.22 to 0.92]	0.778	*g* = −0.09
Change at Week 6	−1.70 (1.53)	−1.60 (1.23)	−0.10 [−0.99 to 0.79]	0.821	*g* = −0.07
*Panel B: presents median (IQR) with Wilcoxon rank-sum testing, Hodges–Lehmann (HL) estimates, and rank-biserial correlation (r*_*rb*_ *). *					
MRS II total score					
Change at Week 12	−5.50 (−9.00, −4.00)	−3.50 (−6.25, −2.00)	−2.00 [−5.00 to 1.00]	0.121	*r*_rb_ = −0.29
Change at Week 6	−4.00 (−7.25, 0.00)	−3.00 (−4.00, −2.00)	−1.00 [−5.00 to 2.00]	0.531	*r*_rb_ = −0.12
Psychological domain score					
Change at Week 12	−1.50 (−3.25, −0.75)	−1.00 (−2.00, 0.00)	−1.00 [−2.00 to 1.00]	0.339	*r*_rb_ = −0.18
Change at Week 6	−1.00 (−3.00, 0.00)	−1.00 (−1.00, 0.00)	0.00 [−2.00 to 1.00]	0.401	*r*_rb_ = −0.15
Urogenital domain score					
Change at Week 12	−1.00 (−3.00, −0.75)	−0.50 (−2.00, 1.00)	−1.00 [−3.00 to 0.00]	0.070	*r*_rb_ = −0.33
Change at Week 6	0.00 (−2.00, 0.00)	0.00 (−1.00, 0.00)	0.00 [−2.00 to 1.00]	0.566	*r*_rb_ = −0.10

Values represent change from baseline (follow-up minus baseline); negative values indicate symptom improvement. Effect sizes are provided for estimation only and should be interpreted cautiously given the sample size

*Abbreviations*:*SD* Standard deviation, *IQR* Interquartile range, *CI* Confidence interval, *HL* Hodges–Lehmann, *r*_*rb*_ rank-biserial correlation

Panel A (mean outcomes):Values are mean (SD). Between-group difference = mean difference (Curcuminoid − Placebo) with 95% CI; P from Welch’s t-test; effect size = Hedges’ g

Panel B (non-normal outcomes):Values are median (IQR). Between-group difference = Hodges–Lehmann (HL) estimate (Curcuminoid − Placebo) with 95% CI; P from Wilcoxon rank-sum test; effect size = rank-biserial correlation (*r*_rb_)

^*^p < 0.05

### Secondary outcomes: MENQOL

For MENQOL total scores, a nominal between-group difference favoring curcuminoids was observed at Week 6 (mean difference in change = − 10.30; 95% CI − 19.81 to − 0.79; *p* = 0.035), but this effect was not sustained at Week 12 (*p* = 0.213) (Table 4, Panel A). The MENQOL vasomotor domain showed a nominal improvement at Week 6 (mean difference in change = − 2.40; 95% CI − 4.03 to − 0.77; *p* = 0.005) (Table 4, Panel A). At Week 12, the primary change-score analysis for the MENQOL vasomotor domain did not reach statistical significance (*p* = 0.149) (Table 4, Panel A). Median (IQR) summaries are presented in Table 4, Panel B.

**Table 4:** Changes in menopause-specific quality of life questionnaire (MENQOL) scores from baseline to weeks 6 and 12

**Outcome / Timepoint**	**Curcuminoid (** ***n*** **=20)**	**Placebo (** ***n*** **=20)**	**Difference** **[95% CI]**	***p*** **-value**	**Effect Size**
*Panel A: presents mean (SD) with Welch’s t-test and Hedges’ g*					
MENQOL Total Score					
Change at Week 12	−24.25 (21.71)	−16.75 (15.02)	−7.50 [−19.50 to 4.50]	0.213	*g* = −0.39
Change at Week 6	−18.55 (17.61)	−8.25 (11.21)	−10.30 [−19.81 to −0.79]	0.035*	*g* = −0.68
Vasomotor Domain					
Change at Week 12^c^	−5.65 (3.62)	−4.15 (2.76)	−1.50 [−3.56 to 0.56]	0.149	*g* = −0.46
Change at Week 6	−4.95 (3.05)	−2.55 (1.85)	−2.40 [−4.03 to −0.77]	0.005*	*g* = −0.93
Physical Domain					
Change at Week 12	−12.50 (13.15)	−8.05 (9.60)	−4.45 [−11.84 to 2.94]	0.230	*g* = −0.38
Change at Week 6	− 9.70 (8.81)	−3.30 (8.39)	−6.40 [−11.91 to −0.89]	0.024*	*g* = −0.73
*Panel B: presents median (IQR) with Wilcoxon rank-sum testing, Hodges–Lehmann (HL) median differences, and rank-biserial correlation (r*_*rb*_ *)*					
Psychosocial Domain					
Change at Week 12	−4.00 (−8.25, −0.75)	−4.00 (−6.50, 1.50)	0.00 [−4.00 to 3.00]	0.849	*r*_rb_ = −0.04
Change at Week 6	−3.50 (−6.75, 0.25)	−2.00 (−4.00, 0.00)	−1.00 [−4.00 to 3.00]	0.522	*r*_rb_ = −0.12
Sexual Domain					
Change at Week 12	−2.50 (−4.00, −1.50)	0.00 (−3.00, 0.00)	−2.00 [−4.00 to 1.00]	0.156	*r*_rb_ = −0.26
Change at Week 6	−2.50 (−3.00, 0.00)	0.00 (−1.00, 0.00)	−2.00 [−3.00 to 0.00]	0.011*	*r*_rb_ = −0.46

Values represent change from baseline (follow-up minus baseline); negative values indicate improvement. Effect sizes are provided for estimation only and should be interpreted cautiously given the sample size

*Abbreviations*:*SD* Standard deviation, *IQR* Interquartile range, *CI* Confidence interval, *HL* Hodges–Lehmann, *r*_*rb*_ rank-biserial correlation

Panel A (mean outcomes): Values are mean (SD). Between-group difference = mean difference (Curcuminoid − Placebo) with 95% CI; P from Welch’s two-sample t-test; effect size = Hedges’ g

Panel B (non-normal outcomes): Values are median (IQR). Between-group difference = HL estimate (Curcuminoid − Placebo) with 95% CI; P from Wilcoxon rank-sum test; effect size = r_*rb*_

^c^Baseline-adjusted sensitivity analysis (ANCOVA) for the MENQOL vasomotor domain at Week 12 is reported in Additional file 1: Table S2

^*^p < 0.05

### Sensitivity and exploratory analyses

Baseline-adjusted sensitivity analyses at Week 12 are presented in Additional file 1: Table S2 as supportive analyses and do not replace the prespecified change-from-baseline analysis. For the primary outcome, baseline-adjusted Poisson regression (IRRs with 95% CIs) was consistent in direction with the primary analysis (IRR = 0.41; 95% CI 0.32–0.52; *p* < 0.001). For secondary outcomes, baseline-adjusted ANCOVA results were generally not statistically significant; the MENQOL vasomotor domain showed a nominal improvement (adjusted mean difference − 2.06; 95% CI − 3.70 to − 0.43; *p* = 0.015), whereas MENQOL total score and other domains were not statistically significant.

### Trial conduct, adherence, and safety

Of 48 women assessed for eligibility, 40 were randomized (83.3%). Retention was 100%, with all participants completing the 12-week intervention and attending scheduled visits. Adherence to the assigned intervention was high and comparable in both groups, with a mean (SD) adherence of 95.3% (2.6) in the curcuminoid group and 94.7% (2.2) in the placebo group, based on returned capsule counts. No participants withdrew or discontinued the intervention. No serious adverse events (SAEs) were reported. One participant in the curcuminoid group experienced a single episode of postmenopausal bleeding, which resolved spontaneously without further intervention.

## Discussion

This randomized, double-blind, placebo-controlled pilot proof-of-concept trial showed a preliminary signal that curcuminoid supplementation was associated with a greater reduction in weekly hot flash frequency than placebo, with between-group separation evident by Week 6 and maintained through Week 12 (prespecified primary outcome). The MENQOL vasomotor domain scores also improved, particularly at Week 6, whereas between-group differences were not consistently observed for other MENQOL domains or overall menopausal symptom severity (MRS II). The intervention was well tolerated with high adherence, supporting further evaluation in an adequately powered confirmatory trial.

This pattern is expected because multidomain instruments capture broader constructs beyond vasomotor symptoms, and this pilot study was not powered to detect changes across all domains [30, 33]. The earlier separation at Week 6 across vasomotor-related outcomes is biologically plausible given established thermoregulatory models of hot flashes and emerging links with oxidative stress and inflammatory signaling in menopause [24, 25, 34]. Curcuminoids have documented antioxidant and anti-inflammatory actions across relevant pathways, providing a mechanistic rationale for symptom improvement [35, 36]. At Week 12, the MENQOL vasomotor between-group estimate was attenuated despite sustained improvement in absolute hot flash frequency. This pattern may reflect time-dependent placebo-related improvement and outcome variability commonly reported in vasomotor symptom trials [37, 38].

Interpretation of secondary outcomes should also consider baseline characteristics: although baseline differences between groups were not statistically significant, the curcuminoid group exhibited numerically higher baseline hot flash frequency (15.55 vs. 13.30 episodes/week) and higher total MENQOL scores (89.80 vs. 83.70) compared with the placebo group (Additional file 1: Tables S1A and S1C). Accordingly, our primary inference emphasized between-group comparisons of change from baseline. Supportive baseline-adjusted sensitivity analyses are reported in Additional file 1: Table S2. Absolute hot flash frequency at follow-up was also lower in the curcuminoid group, supporting the direction of the change-score findings. For the MENQOL vasomotor domain at Week 12, where the estimate appeared sensitive to baseline severity, we presented an exploratory baseline-adjusted ANCOVA as a sensitivity analysis (Additional file 1: Table S2), consistent with recommendations on baseline adjustment and interpretation [39].

The magnitude of symptom reduction should be interpreted relative to baseline severity and the context in which minimal clinically important difference (MCID) thresholds were derived [40, 41]. For instance, neurokinin receptor antagonist trials often enroll women with higher baseline symptom burden (e.g., ~ 7–8 moderate-to-severe hot flashes/day) [42]. In our population with a more moderate baseline symptom profile, the mean between-group difference in weekly hot flash reduction at Week 12 (7.35 episodes/week) corresponds to approximately 1 hot flash/day, which may be below some published daily MCID thresholds derived from populations with more severe symptoms. However, the proportional reduction from baseline in weekly hot flash frequency was substantially larger in the curcuminoid group than in the placebo group (approximately 67% versus 23%, respectively, based on group mean changes), and this pattern was accompanied by improvements in vasomotor-specific quality of life. Together, these findings suggest potential clinical relevance for the symptom severity profile studied. However, as a pilot proof-of-concept trial, effect-size estimates are imprecise and may overestimate true effects; therefore, findings should be interpreted cautiously and confirmed in a larger, adequately powered randomized trial. Our results are broadly consistent with prior trials and meta-analytic evidence suggesting the potential benefit of curcumin/curcuminoids for vasomotor symptoms [13, 15].

The preclinical literature suggests that curcuminoids may act through antioxidant, anti-inflammatory, and neuroregulatory pathways relevant to vasomotor symptoms. Oxidative stress and endothelial dysfunction have been proposed contributors to thermoregulatory instability relevant to hot flashes [34, 43]. Curcumin may modulate oxidative stress through direct scavenging of free radicals and reactive oxygen species (ROS), enhancement of endogenous antioxidant defenses (including superoxide dismutase [SOD], catalase [CAT], and glutathione peroxidase [GPx]), and improvement of overall redox balance [13, 15, 26, 36, 43]. These effects may be mediated in part via Nrf2 activation, which upregulates cytoprotective enzymes including SOD [35, 44] and are supported by clinical reports of increased serum total antioxidant capacity (TAC) and reduced malondialdehyde (MDA) following curcumin supplementation [13]. Curcumin may also improve vascular endothelial function, potentially counteracting age-related vascular decline [9, 36]; this is relevant because estrogen exerts antioxidant effects by stimulating cellular antioxidant enzymes, and this capacity diminishes after menopause [43, 45]. Beyond oxidative stress, menopause is associated with chronic low-grade inflammation, reflected by increased cytokines (e.g., TNF-α, IL-6) and higher C-reactive protein (CRP) [13]. Curcumin has been reported to mitigate inflammatory signaling, including inhibition of nuclear factor kappa B (NF-κB), with downstream reductions in pro-inflammatory mediator expression [46, 47], which may plausibly attenuate systemic and neurovascular inflammation contributing to vasomotor instability. Finally, estrogen withdrawal has been linked to altered serotonergic and noradrenergic tone in VMS pathogenesis [39], and experimental ovariectomized models suggest curcumin may influence neurotransmitter-related pathways involved in thermoregulation [12]. Collectively, these pathways provide a biologically plausible rationale for the observed symptom improvement. However, because biochemical markers were not assessed in this trial, any link between these mechanisms and our observed clinical findings remains speculative, and future adequately powered trials incorporating biomarker endpoints are needed.

### Strengths and limitations

This pilot trial demonstrated several strengths. Its randomized, placebo-controlled, and double-blind design supports internal validity. The use of validated outcome instruments (MRS II and MENQOL), structured adherence monitoring, and excellent participant retention ensured high-quality data. The inclusion of both symptom diaries and quality-of-life questionnaires enabled multidimensional assessment of intervention effects. As a pilot proof-of-concept trial, it also demonstrated strong trial conduct (no loss to follow-up, high adherence) and good tolerability, with no serious adverse events. Notably, this trial uniquely used curcuminoid monotherapy at a standardized dose (1000 mg/day) for 12 weeks, thereby isolating its specific effects on VMS. Furthermore, it contributes valuable region-specific data from Southeast Asian populations, who are often underrepresented in menopause studies.

However, several limitations warrant consideration. As a pilot proof-of-concept trial, the sample size was not powered to detect small-to-moderate between-group effects, particularly for secondary outcomes; therefore, non-significant findings should not be interpreted as evidence of no effect, and observed effects should be considered preliminary and used to inform the design of a future adequately powered confirmatory trial. The study population was heterogeneous (age range 41–65 years; natural vs. surgical menopause), which may have increased variability and reduced power to detect between-group differences. While this reflects routine clinical practice, future larger trials should consider stratification (e.g., by menopause type and baseline VMS severity) or narrower eligibility criteria. Although we reported standardized effect sizes to support estimation and future sample size planning in this pilot proof-of-concept trial, these estimates should be interpreted cautiously because effect sizes from small pilot studies may be unstable and may overestimate the true magnitude of effect. Accordingly, we avoid qualitative labels (e.g., ‘large’) and present effect sizes as preliminary, hypothesis-generating measures. In addition, multiple secondary outcomes were assessed across domains and time points without formal adjustment for multiple comparisons, increasing the risk of Type I error. Therefore, secondary outcome findings should be interpreted as exploratory and hypothesis-generating rather than confirmatory. This is particularly relevant for findings with p-values > 0.01. Future definitive trials should prespecify multiplicity-control strategies, such as hierarchical testing, or apply appropriate adjustments to validate these preliminary signals. The 12-week study duration limits conclusions regarding long-term efficacy and safety. The absence of biochemical assessments (e.g., oxidative stress or inflammatory markers) restricted mechanistic interpretation. The study focused specifically on vasomotor symptoms, so findings may not be generalizable to other menopausal domains. Although trial registration was retrospective, the protocol was approved prior to enrolment and the registered primary outcome and target sample size were reviewed and found consistent with this manuscript; nevertheless, retrospective registration remains a limitation. Interpreting the overall clinical importance of findings in menopausal symptom studies remains challenging because diverse outcome scales and metrics complicate the derivation and application of precise minimal clinically important differences (MCIDs) across domains. Overall, these findings should be interpreted as preliminary and hypothesis-generating and used to inform the design of larger, adequately powered, prospectively registered confirmatory trials.

### Clinical implications and future research

Curcuminoid supplementation may warrant further evaluation as a well-tolerated, non-hormonal approach for managing VMS in postmenopausal women, particularly for those who are ineligible for or decline hormone therapy; however, efficacy requires confirmation.

Future studies should focus on multicenter trials with larger sample sizes, extended follow-up periods, and inclusion of biochemical endpoints such as oxidative stress markers, inflammatory cytokines, and hormonal profiles to elucidate the underlying mechanisms of action. Comparative trials with other non-hormonal agents (e.g., SSRIs, gabapentin), dose optimization studies, and long-term safety assessments are also warranted to better define curcuminoid’s role in evidence-based menopause care. Additionally, real-world effectiveness studies through observational or pragmatic trials would further clarify its value in broader clinical and community contexts.

## Conclusion

In conclusion, this randomized, double-blind, placebo-controlled pilot proof-of-concept trial observed a preliminary signal that 12-week curcuminoid supplementation significantly reduced weekly hot flash frequency compared with placebo and was well tolerated. A preliminary signal toward improved vasomotor-related quality of life was also observed, particularly at Week 6. Given the pilot design and modest sample size, these findings are hypothesis-generating and require confirmation in larger, adequately powered randomized trials, ideally incorporating biomarker assessments.

## Supplementary Information

Supplementary Material 1.

## Data Availability

The study protocol, statistical analysis plan, and datasets generated and analyzed during the current study are available from the corresponding author upon reasonable request. The study protocol, statistical analysis plan, and deidentified participant data are available from the corresponding author upon reasonable request.

## Abbreviations

ACOG: American College of Obstetricians and Gynecologists
BMI: Body Mass Index
DM: Diabetes Mellitus
FSH: Follicle–Stimulating Hormone
GRAS: Generally Recognized as Safe
HT: Hormone Therapy
HTN: Hypertension
IL: 6 Interleukin–6
MENQOL: Menopause–Specific Quality of Life Questionnaire
MRS II: Menopause Rating Scale II
MDA: Malondialdehyde
ROS: Reactive Oxygen Species
RCT: Randomized Controlled Trial
SOD: Superoxide Dismutase
SSRI: Selective Serotonin Reuptake Inhibitor
SNOSE: Sequentially Numbered, Opaque, Sealed Envelope
TCTR: Thai Clinical Trials Registry
THC: Tetrahydrocurcumin
TNF: α–Tumor Necrosis Factor–alpha
VMS: Vasomotor Symptoms
