# Supplementary material for: Curcuminoid supplementation for vasomotor symptoms in postmenopausal women: a pilot, randomized, double-blind, placebo-controlled proof-of-concept trial
Source: BMC Complement Med Ther. 2026 Mar 24;26:164. doi: 10.1186/s12906-026-05358-7 (PMC13134235; doi:10.1186/s12906-026-05358-7)
Supplement: Supplementary file 1 — Supplementary Material 1. [file 12906_2026_5358_MOESM1_ESM.docx]

**Supplementary Table S1. Absolute scores at each timepoint (Baseline/Week 6/Week 12)**

**S1A. Weekly hot flash frequency (episodes/week)**

| **Timepoint** | **Curcuminoid (n=20)**  **Mean (SD)** | **Placebo (n=20)**  **Mean (SD)** |
| --- | --- | --- |
| Baseline | 15.55 (8.30) | 13.30 (6.01) |
| Week 6 | 6.15 (4.17) | 10.85 (5.36) |
| Week 12 | 5.20 (3.76) | 10.30 (5.31) |

**S1B. MRS II absolute scores**

| **Outcome** | **Timepoint** | **Curcuminoid (n=20)**  **Mean (SD)** | **Placebo (n=20)**  **Mean (SD)** |
| --- | --- | --- | --- |
| MRS II total score | Baseline | 10.95 (5.03) | 10.25 (4.86) |
| MRS II total score | Week 6 | 6.95 (4.44) | 7.25 (4.41) |
| MRS II total score | Week 12 | 5.45 (3.72) | 6.75 (4.34) |
| MRS II somato-vegetative domain score | Baseline | 4.15 (2.19) | 4.15 (1.86) |
| MRS II somato-vegetative domain score | Week 6 | 2.45 (1.55) | 2.55 (1.37) |
| MRS II somato-vegetative domain score | Week 12 | 1.90 (1.24) | 2.05 (1.33) |
| MRS II psychological domain score | Baseline | 4.10 (2.18) | 4.05 (2.14) |
| MRS II psychological domain score | Week 6 | 3.10 (1.94) | 3.05 (1.76) |
| MRS II psychological domain score | Week 12 | 2.60 (1.60) | 3.05 (1.76) |
| MRS II urogenital domain score | Baseline | 2.70 (1.84) | 2.05 (1.76) |
| MRS II urogenital domain score | Week 6 | 2.70 (1.84) | 2.05 (1.76) |
| MRS II urogenital domain score | Week 12 | 1.70 (1.42) | 2.00 (1.56) |

**S1C. MENQOL absolute scores**

| **Outcome** | **Timepoint** | **Curcuminoid (n=20)**  **Mean (SD)** | **Placebo (n=20)**  **Mean (SD)** |
| --- | --- | --- | --- |
| MENQOL total score | Baseline | 89.80 (23.42) | 83.70 (21.91) |
| MENQOL total score | Week 6 | 71.25 (23.74) | 75.45 (20.72) |
| MENQOL total score | Week 12 | 65.55 (26.42) | 66.95 (22.43) |
| MENQOL vasomotor domain score | Baseline | 11.10 (3.85) | 12.10 (3.37) |
| MENQOL vasomotor domain score | Week 6 | 6.15 (1.60) | 9.55 (3.24) |
| MENQOL vasomotor domain score | Week 12 | 5.45 (1.79) | 7.95 (3.78) |
| MENQOL psychosocial domain score | Baseline | 26.00 (9.14) | 25.05 (8.61) |
| MENQOL psychosocial domain score | Week 6 | 22.50 (8.69) | 23.05 (8.39) |
| MENQOL psychosocial domain score | Week 12 | 22.00 (9.67) | 21.05 (8.35) |
| MENQOL physical domain score | Baseline | 46.25 (14.61) | 42.60 (13.29) |
| MENQOL physical domain score | Week 6 | 36.55 (14.15) | 39.30 (12.77) |
| MENQOL physical domain score | Week 12 | 33.75 (16.13) | 34.55 (13.51) |
| MENQOL sexual domain score | Baseline | 6.45 (4.07) | 3.95 (3.98) |
| MENQOL sexual domain score | Week 6 | 4.30 (3.67) | 3.70 (3.84) |
| MENQOL sexual domain score | Week 12 | 3.95 (3.77) | 3.95 (3.98) |

**Note (S1): Values are presented as mean (SD) of absolute scores at each visit and are provided for descriptive purposes only. Between-group comparisons and p-values for the primary outcome are reported in Table 2 using change-from-baseline analyses.**

**Supplementary Table S2. Baseline-adjusted analyses at Week 12 (sensitivity analysis)**

| **Outcome** | **Method** | **Adjusted Effect Estimate (95% CI) ᵃ** | **P-value** |
| --- | --- | --- | --- |
| **Primary Outcome** |  |  |  |
| Hot Flash Frequency | Poisson Regression | IRR = 0.41 (0.32 to 0.52) | <0.001* |
| **Secondary Outcomes: MENQOL score** | | | |
| Vasomotor Domain | ANCOVA | MD = −2.06 (−3.70 to −0.43) | 0.015* |
| Psychosocial Domain | ANCOVA | MD = 0.94 (−2.40 to 4.28) | 0.571 |
| Physical Domain | ANCOVA | MD = −2.94 (−9.39 to 3.51) | 0.362 |
| Sexual Domain | ANCOVA | MD = −1.00 (−3.18 to 1.18) | 0.358 |
| Total Score | ANCOVA | MD = −5.13 (−15.94 to 5.68) | 0.343 |
| **Secondary Outcomes: MRS II Score** | | | |
| Somato-vegetative Domain | ANCOVA | MD = −0.24 (−1.24 to 0.77) | 0.635 |
| Psychological Domain | ANCOVA | MD = −0.19 (−1.45 to 1.07) | 0.762 |
| Urogenital Domain | ANCOVA | MD = −0.90 (−2.20 to 0.39) | 0.164 |
| Total Score | ANCOVA | MD = −1.75 (−4.48 to 0.99) | 0.203 |

Notes: ᵃ Adjusted Effect Estimate represents:

Incidence Rate Ratio (IRR) for hot flash frequency (derived from Poisson regression with log link: *Week 12 Frequency ~ Group + Baseline Frequency*). An IRR < 1.0 indicates a greater reduction in the curcuminoid group.

Adjusted Mean Difference (MD) for MENQOL and MRS II scores (derived from ANCOVA: *Week 12 Score ~ Group + Baseline Score*). Negative values indicate greater symptom reduction in the curcuminoid group.

CI, confidence interval; ANCOVA, analysis of covariance; MENQOL, Menopause-Specific Quality of Life; MRS II, Menopause Rating Scale II.

* p < 0.05.
